# 

**Published:** 1887-09

**Authors:** 


### EDITORIALS AND MISCELLANEOUS.

### TO OUR SUBSCRIBERS.

The fact that you do not receive your Record is no evidence that it has not been mailed. When you fail to receive it notify us at once, and do not wait till your subscription expires and the bill is presented to inform us of your disappointment, and consequent refusal to pay for your journal. Should you change your residence and post-office, should the Government alter or suppress the mail delivery in your section, give us notification thereof. We attend strictly to all the details of our business at *this end* of the line. We are not presumed to know either by instinct or intuition any irregularity in the mail service. Should you not, therefore, receive the Record by reason of these circumstances, you and you alone are to be blamed, not we. Is it too much or unreasonable to ask you to keep a little watch at *your end* of the line? Let us have fair play.

RECEIPTED.

1886—Drs. J. L. Hamilton, Thos. A. Phillips, R. R. Mayo, K. L. Law.

1887—Drs. M. W. Fowler, J. R. Johnson, G. w. Earle, Jas. Jackson, Wm. Martin, E. E. Batey, M. L. Boyd.

1888—Drs. M. R. Toland, to July; Jas. Jewett, R. A. Mazill, E. L. Hammond.

